# Wetland Changes and Their Relation to Climate Change in the Pumqu Basin, Tibetan Plateau

**DOI:** 10.3390/ijerph18052682

**Published:** 2021-03-07

**Authors:** Yihao Zhang, Jianzhong Yan, Xian Cheng, Xinjun He

**Affiliations:** 1 College of Resources and Environment, Southwest University, Chongqing 400715, China; zhangyhswu@126.com (Y.Z.); yanjzswu@126.com (J.Y.); hxjswu@126.com (X.H.); 2 State Cultivation Base of Eco-Agriculture for Southwest Mountainous Land, Southwest University, Chongqing 400715, China

**Keywords:** wetland changes, climate change, the Pumqu River Basin, Tibetan Plateau, reclamation

## Abstract

Wetland ecosystems play one of the most crucial roles in the world. Wetlands have the functions of ecological water storage, water supply, and climate regulation, which plays an indispensable role in global environmental security. The Pumqu River Basin (PRB) is located in an area with extremely vulnerable ecological environment, where climate change is obvious. Understanding wetland distribution, changes and causes in the PRB are of great importance to the rational management and protection of wetlands. Using the Landsat series satellite images, wetlands of this area in 2000, 2010, and 2018 were extracted. The results showed that (1) there were obvious regional differences in wetland types and their distribution patterns in the basin. Wetlands were mainly distributed in areas with slopes less than 12° and at elevations between 4000 m and 5500 m. (2) During the past 20 years, the wetland area in the basin decreased, and the changing trend of wetlands was different. Palustrine wetlands decreased tremendously, riverine and lacustrine wetlands first decreased and then increased, while floodplain wetlands first increased and then decreased. Palustrine wetlands were reclaimed to cultivated land, but the proportion of reclamation is small. (3) Climate dominated wetland changes in the PRB. The changes in riverine and lacustrine wetlands were mainly affected by the warm-season average temperature, the change in palustrine wetlands was mainly related to the annual precipitation and the warm-season average temperature, and the change in floodplain wetlands was related to the warm-season precipitation. To achieve sustainable development, the government plays a guiding role and actively formulates and implements wetland protection policies, such as restricting or prohibiting grazing on wetlands, which play an important role in wetland protection and restoration.

## 1. Introduction

Wetlands usually have to a unique natural complex of species that interact with the environment at the junction of land and water, with the overall water level being close to or at the surface [1]. Wetlands are one of the most important ecosystems, and play a key role in regulating runoff, mitigating floods, and improving water quality. They also provide the necessary living environment for many animals and plants, maintain ecosystem stability, and protect biodiversity [2,3]. However, in the past several decades, due to human activities increasing and climate change becoming more intense, the rapid reduction and degradation of wetlands has become a global phenomenon [4]. Statistics show that since 1700, wetland loss in the world has probably reached 87%. Since 1900, wetland loss has reached 64–71%, which is 3.7 times faster than before [5]. Wetland ecosystems have become one of the most threatened ecosystems in the world [6]. Wetlands are mainly distributed in Asia, South America, and North America, which account for approximately 80% of the total wetland area in the world [7,8]. The Pantanal, distributed in South America, is the largest natural wetland in the world, with an area of 15 × 10^4^ km^2^ [9]. Destroyed by human activities such as fire, deforestation and excessive agricultural development, the Pantanal wetlands are seriously threatened [10,11]. In September 2019, the area burned by fire reached 321.1 km^2^ [12]. The wetland area in Canada accounts for 51% of that in North America. Southern Ontario, with the largest population distribution in Canada, has had a wetland loss rate of more than 80% [13]. In addition, 32% of the tidal marshes in the Saint Lawrence Estuary have been reclaimed as cultivated land [13]. From a global perspective, Asselen et al. [4] conducted a meta-analysis of 105 wetland change cases around the world and found that agricultural development is the main direct cause of wetland changes and that economic growth and population density are the most common potential forces. Hu et al. [8] also emphasized that on a global scale, human activities are the main causes of wetland loss and degradation.

Wetland loss is the highest in Asia, and it is a key area of wetland degradation in the world [8]. China is the country with the largest wetland area in Asia, with a wetland area of approximately 45 × 10^4^ km^2^ (2015) [14]. Wetland conditions in China have an important impact on wetland changes in Asia. By the end of 2020, China had established 899 national wetland parks (http://www.mnr.gov.cn/ (accessed on 1 February 2021)). However, China’s natural wetlands decreased by 2.63 × 10^4^ km^2^ between 1990 and 2010, resulting in wetland loss of approximately 7%, of which approximately 60% (1.58 × 10^4^ km^2^) of the area was converted to agricultural land [15]. Northeast China and the Tibetan Plateau have the most widely distributed wetlands in China, accounting for 50% of the total wetland area in China [14]. Current research has found that wetland changes in Northeast China are mainly affected by human activities such as agricultural reclamation, fires, engineering construction, and climate change [16]. Mao et al. [17] compared the wetland changes between China and Russia in the Amur River Basin and found that wetlands on the Chinese side were mainly affected by agricultural reclamation, while wetlands on the Russian side were mainly affected by climate change due to the small population and underdeveloped economy. Because the Tibetan Plateau is cold and has a high altitude, the population is small and concentrated in cities and towns; thus, economic development and human activity intensity are low [18]. Compared with the wetlands in Northeast China, the Tibetan Plateau wetlands are much less affected by human activities. The climate of the Tibetan Plateau is affected by the Indian monsoon, East Asian monsoon, and westerlies, and the climate change in this region is more intense than that in other regions [19]. Wetland changes on the Tibetan Plateau may be more closely related to climate. Climate change affects wetland ecosystems mainly through the changes in temperature and precipitation [20]. Studies have shown that precipitation changes are the main cause of wetland changes in the source areas of the Yellow River and the Yangtze River [21,22]. However, the situation is different in the eastern part of the Tibetan Plateau. The Zoige Plateau has the largest palustrine wetland on the Tibetan Plateau [23]. Agricultural and animal husbandry activities (e.g., reclamation, ditch construction, overgrazing) have caused serious hydrological losses of palustrine wetlands, and these activities are the main reason for wetland degradation [24,25,26].

With increasing awareness of wetland ecosystem services, the value of wetlands has received increasing attention. Many countries, including China, have taken measures to protect wetlands by developing relevant laws and policies and establishing nature reserves [27,28]. Therefore, understanding wetland changes is important for the formulation of national policies and regulations, environmental protection, and ecological restoration. With the advantages of wide coverage, real-time information acquisition, convenient collection, and periodicity, remote sensing technology has been widely used in the survey and identification of wetland resources for nearly 30 years. Although computer automatic classification is a fast method for wetland extraction, regional limitations and low accuracy make visual interpretation the best choice [29]. Li et al. [21] extracted six wetland types in Madou County by visual interpretation and found that the impact of precipitation on wetlands was more prominent. Visual interpretation is more conducive to extracting different wetland types according to local hydrological and geomorphic characteristics and revealing the impacts of environmental conditions on wetlands.

The Pumqu River Basin (PRB) is located in the alpine region on the north side of the Himalayas and is the core area of the Everest Nature Reserve. The PRB is surrounded by mountains, which makes it difficult for warm and humid air to enter, resulting in little annual rainfall and an extremely fragile ecological environment coupled with large evaporation and severe drought [30,31]. In the basin, wetlands are widely distributed, and there are breeding grounds for rare wetland birds, such as the Black Necked Crane, which is a first-class national protected animal [32]. In addition, located on the Tibetan Plateau, the PRB is sensitive to global climate change, and is located in the most significant region of global warming in the same period. As one of the most significant regions of climate change, the impacts of climate factors on local alpine wetlands are more pronounced [33]. Additionally, the cultivated land area of Tingri, Dinggye, and Nyalam Counties increased by 13.86 km^2^ from 2000 to 2018 (Tibet Statistical Yearbook), and the field investigation of the research group in the PRB from July to August 2018 found that there was a phenomenon of farmers reclaiming palustrine wetlands to cultivated land, but the specific situation is unknown. In previous studies, there was relatively little concern about wetland resources in the PRB, and wetland distribution and change data were lacking. This paper explores the changing laws of alpine wetlands and their links with climate change, and clarifies the impact of human activities (cultivated land reclaimed from palustrine wetlands) to provide a reference and basis for the protection and restoration of alpine wetland ecosystems and to maintain the sustainable development of ecologically vulnerable areas and global ecological security.

The specific objectives of this study are to (a) master the spatial distribution and changes of different wetland types; (b) compare the wetland changes in three time horizons; and (c) explore the relationship between wetland changes and climate change.

## 2. Materials and Methods

### 2.1. Study Area

**Figure 1:**
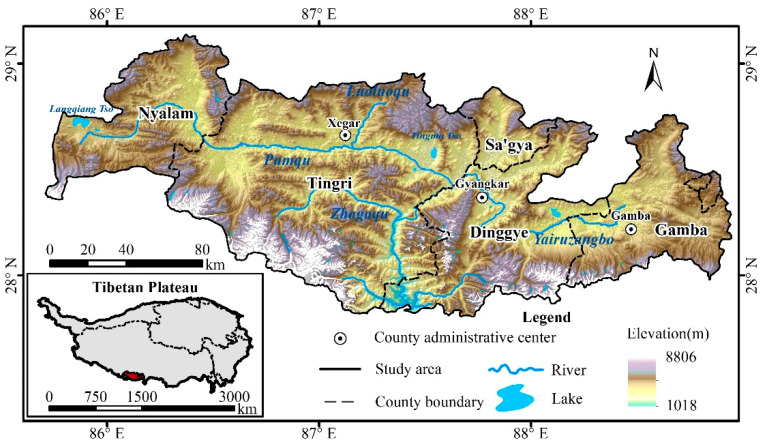
Location of the Pumqu River Basin (PRB).

The PRB, located in the southwestern part of the Tibetan Plateau, China (Figure 1), is bounded by the Himalayas to the south, the Kailas Range to the north, the Paiku Co to the west, and the Kangcheda Snow Mountain Group to the east. The PRB is in the upper reaches of the Koshi River, an important transnational river in South Asia, and is also the core area of the Everest Nature Reserve. The geographical range is 85°38′~88°57′ E and 27°49′~29°05′ N, covering parts of 5 counties, including Nyalam, Tingri, Dinggye, Gamba and Sa’gya (Figure 1). It covers an area of 25,018 km^2^ with an average elevation of 5259 m. The Foehn effect is very pronounced in the region. The annual average temperature of the PRB in 1997–2017 fluctuated between 3.5 °C and 4.5 °C, and the average temperature of the warm season (June–September) ranged from 10.5 °C to 11 °C. The temperature is below 0 °C from January to March, November and December. The annual precipitation is between 800 mm and 1100 mm, and the warm-season precipitation accounts for 50–70% of the annual precipitation. The local crops are mainly barley, wheat and canola. Livestock includes yaks, cattle-yaks, cows, horses, and sheep. The PRB has abundant wetland resources, and palustrine wetlands are the most common. The rivers mainly include the Pumqu River, the Zhagaqu River, and the Yairuzangbo River. The largest lakes in the area are Langqiang Tso and Tingmo Tso. The land cover types in the region are wetlands, cultivated land, grasslands, woodlands, glaciers and bare land. In 2015, the total population of the basin was approximately 92,600 and the population density was approximately 3.7 people/km^2^.

### 2.2. Data Source

#### 2.2.1. Remote Sensing Data

The study used Landsat 5 Thematic Mapper (TM) satellite images from 2000 and 2010 and Landsat 8 Operational Land Imager (OLI) satellite images from 2018. All images had a resolution of 30 m and were downloaded from the US Geological Survey website (https://earthexplorer.usgs.gov/ (accessed on 30 October 2018)). Although the vegetation of the wetlands grows lush in summer, due to the special geographical location and environment, there is a large amount of cloud cover in images during this season. Images with less than 10% cloud cover were selected for use in this study (Table 1).

#### 2.2.2. Topographic and Meteorological Data

ASTER GDEM data with a spatial resolution of 30 m, downloaded from the USGS website, were used in this study. According to the geographical range of the study area, we selected 8 scenes of the DEM, and their respective numbers were N29E086, N28E085, N28E086, N28E087, N28E088, N27E086, N27E087, and N27E088. The DEM data were mosaicked and cut to obtain the DEM map, and the slope was extracted by the DEM.

Temperature and precipitation data from 1997 to 2017 at the Tingri and Nyalam Weather Stations were downloaded from the China Meteorological Data Network (https://data.cma.cn/ (accessed on 30 October 2018)). The raw data from the website are the daily temperature and precipitation data from the two weather stations. We used Excel to process these data. The daily temperature data from a year were averaged to obtain the average annual temperature. The daily temperatures from June to September were averaged to obtain warm-season average temperatures. Annual precipitation was obtained by summing the daily precipitation in a year. Warm-season precipitation was obtained by summing the daily precipitation from June to September.

### 2.3. Methods

#### 2.3.1. Alpine Wetland Classification and Extraction

There is no uniform standard for wetland classification in previous studies. Muro et al. [34] divided the wetlands of the Rhone River Delta, France, into marshlands, temporal water bodies and salt marshes. Tong et al. [35] extracted wetlands from the Three-River Headwaters Region, China into two first categories: Water bodies and swamps, with the water bodies then being divided into streams and rivers, lakes, reservoirs and ponds. Zhang et al. [36] classified the wetlands in the Damqu Basin, the source area of the Yangtze River, into three categories: Riverine, lacustrine and palustrine. There are certain differences in the wetland classification according to the characteristics of the research areas. According to the hydrologic condition of the PRB and the soil and vegetation characteristics of wetlands, and referring to the alpine wetland classification system developed by relevant research [36,37], especially the wetland classification system in the Mount Everest Region [38], the wetland classifications in the PRB were determined as shown in Table 2.

After the establishment of the alpine wetland classification system and interpretation keys, wetland distribution and area were obtained through visual interpretation from remote sensing images. Fifteen Landsat images were mosaicked and cut in ENVI 5.3 to generate the study area satellite images in 2000, 2010, and 2018. A color composite of the near-infrared band (red color), red band (green color), and green band (blue color) was displayed on screen for visual interpretation and is considered to be the best composition of bands for identifying wetlands. Visual interpretation was performed in ArcGIS 10.2. A small number of wetlands and grasses, which are prone to confusion, were identified by visual interpretation and verified in Google Earth. To explore the situation of farmers reclaiming palustrine wetlands to cultivated land in the PRB, cultivated land data in 2010 and 2018 were obtained by visual interpretation. Because this paper focuses on wetland changes, cultivated land distribution and area data were not listed. The interpretation accuracy and the Kappa coefficient were both greater than 0.95.

#### 2.3.2. Grey Correlation Analysis

Grey correlation analysis is a quantitative comparative analysis of the development trend among various factors. The correlation degrees among many factors can be analyzed by comparing the geometric relations of the statistical data series. This method does not require too many samples, so it is more suitable to analyze the correlation between small samples and multiple factors.

The comprehensive factors of the climate change impact on wetlands have extensive grey character, that is, incomplete information and uncertainty. It is suitable to use grey system theory to study the comprehensive evaluation. Grey correlation analysis has been widely used in the study of the relationship between land changes (including wetland changes) and environmental factors [39,40,41]. The calculation formula of the grey correlation degree is as follows:

(1) $$r\left(x_{0}{,\;x}_{i}\right)=\frac{1}{n}\overset{n}{\underset{k=1}{\sum }}r\left(x_{0}\left(k\right){,\;x}_{i}\left(k\right)\right)$$

(2) $$r\left(x_{0}{(k),\;x}_{i}\left(k\right)\right)=\frac{\underset{i}{\min}\underset{k}{\min}\left|x_{0}(k)\;-{\;x}_{i}\left(k\right)\right|+\rho \underset{i}{\max}\underset{k}{\max}\left|x_{0}(k)\;-{\;x}_{i}\left(k\right)\right|}{\left|x_{0}{(k)\;-\;x}_{i}\left(k\right)\right|+\rho \underset{i}{\max}\underset{k}{\max}\left|x_{0}(k)\;-{\;x}_{i}\left(k\right)\right|}$$

where x_0_ is the reference sequence, x_i_ is the comparison sequence, “ρ” is the resolution coefficient, and “ρ” ∈ [0,1], x_0_(k), and x_i_(k) are the number of points k of x_0_ and x_i_ respectively. In this study, x_0_ represents the wetland area, x_i_ represents the climate data, including temperature and precipitation, and “ρ” is 0.5 according to the related research [39,40,42].

Wetland changes were related to climate in the previous period. Therefore, we referred to Li et al. [43]. Three years of meteorological data (1997, 1998, 1999 meteorological data corresponding to the wetland distribution data in 2000; 2007, 2008, 2009 meteorological data corresponding to the wetland distribution data in 2010; 2015, 2016, 2017 meteorological data corresponding to the wetland distribution data in 2018) were selected to calculate the mean value, and then we obtained relevant meteorological data for the corresponding years of wetland distribution. After the normalization of wetland area and climate data, the grey correlation was calculated.

## 3. Results

### 3.1. Wetland Distribution and Change Characteristics

#### 3.1.1. Wetland Composition and Changes

**Figure 2:**
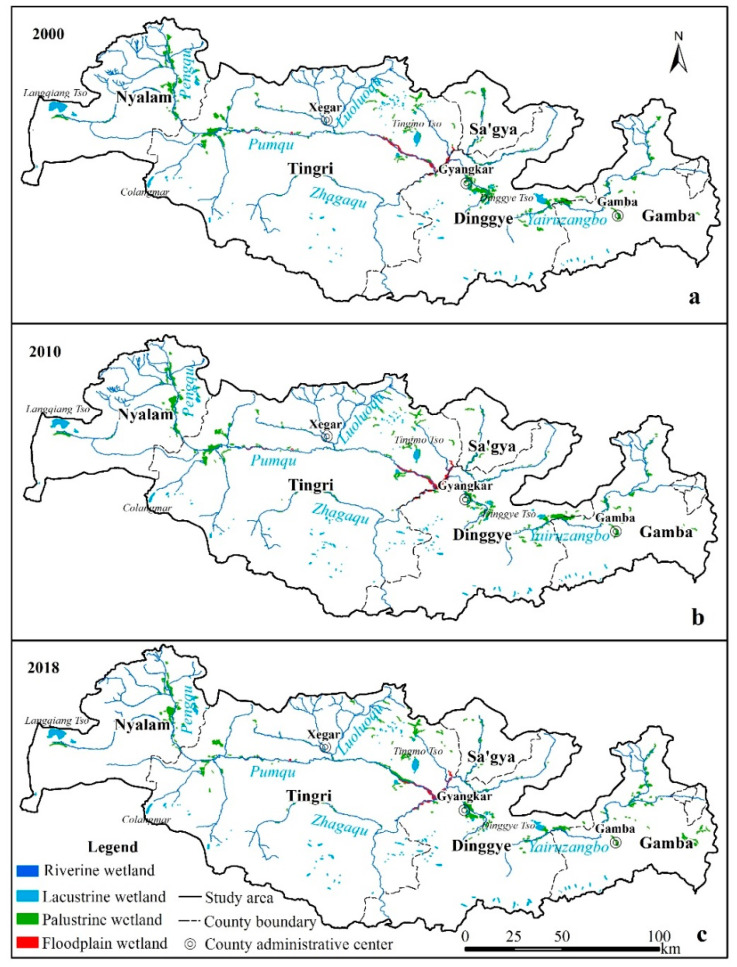
Wetland distribution in the PRB in 2000 (**a**), 2010 (**b**), and 2018 (**c**).

Through the visual interpretation of remote sensing images, the distribution of wetlands in the PRB in 2000, 2010, and 2018 was obtained (Table 3, Figure 2a–c). In 2000, 2010 and 2018, the total wetland area of the PRB was 479.93 km^2^, 458.31 km^2^, and 459.71 km^2^, accounting for 1.92%, 1.83%, and 1.84% of the basin area, respectively. The wetland of the PRB was mainly palustrine wetland, followed by riverine wetland and lacustrine wetland. In 2018, the areas of palustrine wetlands, riverine wetlands, lacustrine wetlands, and floodplain wetlands were 205.83 km^2^, 132.76 km^2^, 92.89 km^2^, and 28.23 km^2^, which accounted for 44.77%, 28.88%, 20.21%, and 6.14% of the total wetland area, respectively (Table 3).

From 2000 to 2018, the wetlands, mainly the palustrine wetlands, in the PRB degraded. The total wetland area was reduced by 20.22 km^2^, with a decrease of 1.12 km^2^·year^−1^ and a degradation rate of 4.21%. Among them, palustrine wetlands were the most seriously degraded, with an area of 23.11 km^2^. The floodplain wetlands showed slight degradation, while the riverine and lacustrine wetland areas increased. Floodplain wetlands had the highest change rate, reaching 11.25%, followed by palustrine wetlands, which decreased by 10.09%. Lacustrine wetlands increased by 5.83%, and riverine wetlands changed very little (1.03%) (Table 3).

#### 3.1.2. Horizontal Distribution Characteristics of Wetlands

Alpine wetlands were widely distributed in the PRB and were dominated by palustrine wetlands, which were distributed along the sides of the riverine wetlands. In terms of geographical location, palustrine wetlands were distributed in the central and northern parts of the PRB and were mainly distributed on both sides of the Pengqu, Pumqu, and Yeru Zangbo Rivers, and scattered around some lacustrine wetlands, such as Langqiang Tso, Tingmo Tso, and Dinggye Tso. In terms of administrative districts, Tingri and Dinggye Counties had the largest palustrine wetland areas, but Gamba County had the highest palustrine wetland rate (in the unit area, palustrine wetlands accounted for the highest proportion in Gamba County) (Table 4).

The riverine wetland mainly included Pumqu, Yeru Zangbo, Zhagaqu, Luoluoqu, and Pengqu. The source of Pumqu is near Langqiang Tso in Nyalam County. It flows from west to east through Tingri County and turns south at the junction of Tingri and Dinggye Counties. Yeru Zangbo originates in Gamba County, flows through Dinggye County from east to west, and merges into Pumqu at the junction of Tingri and Dinggye Counties. Zhagaqu originates in southern Tingri County and merges into Pumqu in Tingri Counties from west to east. Luoluoqu originates in northern Tingri County and merges into Pumqu from north to south in southeastern of Xegar town. Pengqu originates in northern Nyalam County and merges into Pumqu in Nyalam County from north to south. In terms of administrative districts, Tingri and Nyalam Counties have the largest riverine wetland area, and Nyalam County has the highest riverine wetland rate.

In terms of geographical location, lacustrine wetlands are mainly distributed in the north and south, and glacial lakes are mainly distributed in the south. The southern boundary of the PRB is the Himalayas. Glaciers and snow mountains, including Mount Everest, are distributed along the southern boundary. Therefore, lacustrine wetlands in the south are mostly glacial lakes formed by melting glaciers and snow water. In terms of administrative districts, lacustrine wetlands are mainly distributed in Tingri and Nyalam Counties. Nyalam County has the highest lacustrine wetland rate. In 2018, large lacustrine wetlands in the basin included Langqiang Tso (23.66 km^2^), Tingmo Tso (10.94 km^2^), Dinggye Tso (6.23 km^2^) and Colangmar (3.96 km^2^). The first three lacustrine wetlands were distributed in the western, central and eastern parts of the PRB. Colangmar is a typical glacier dammed lake, located at the end of the glacier in the southwest. There were 14 lacustrine wetlands larger than 1 km^2^, and 6 of them were glacial lakes in 2018. Judging the nameless glacial lake was based on the location of lacustrine wetlands and glaciers in the satellite images.

Floodplain wetlands were mainly distributed on both sides of the river channel, including Tingri and Dinggye Counties. Tingri County has the highest floodplain wetland area and rate. Large patches of floodplain wetlands were concentrated in river bifurcations (Figure 2). Floodplain wetlands are most concentrated in the place where Yeru Zangbo merges into Pumqu. Because Yeru Zangbo is the largest tributary of Pumqu, when it merges into Pumqu, the water flow on both sides is large, which impacts both banks and forms a floodplain, thus developing into floodplain wetlands [44].

#### 3.1.3. Vertical Distribution Characteristics of Wetlands

**Figure 3:**
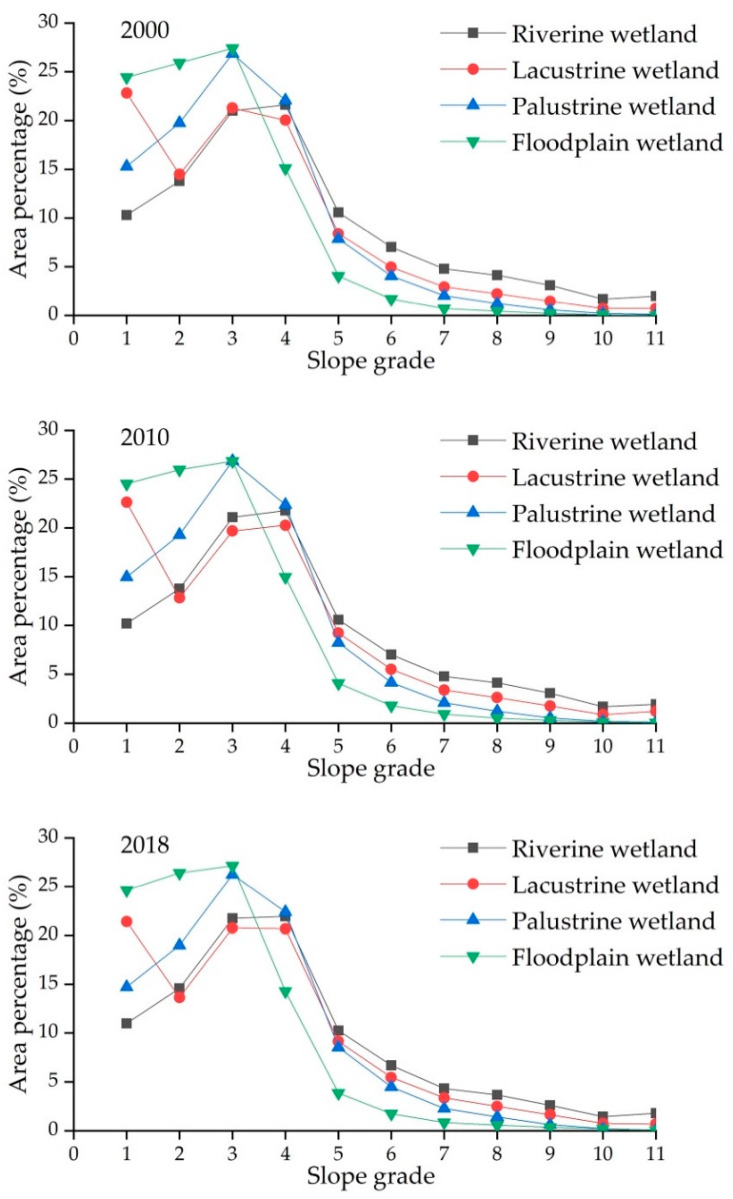
Slope characteristics of wetlands in the PRB.

According to the existing slope file, the slope was divided into 11 grades (Table 5). The elevation was divided at 500-m intervals. The slope, elevation and wetland distribution data in 2000, 2010, and 2018 were superimposed and analyzed to obtain the vertical distribution characteristics of wetlands in the PRB. The results showed that there was a strong slope differentiation among the wetlands (Figure 3). Wetlands were mainly distributed in the region where the slope was less than 12°. These lower slope regions have low terrain and good water accumulation conditions, which are ideal wetland development areas. For the water wetlands, 66–70% of riverine wetlands and 75–79% of lacustrine wetlands were distributed in the region where the slope was less than 12°. A total of 82–84% of palustrine wetlands and 92–93% of floodplain wetlands were distributed in areas with slopes less than 12°. Riverine and lacustrine wetlands were less affected by slope factors than palustrine and floodplain wetlands. Riverine wetlands need to be kept in a certain slope area to maintain a certain flow speed. Compared with riverine and lacustrine wetlands, palustrine and floodplain wetlands have a considerable dependence on topographic conditions. The hygrophyte community in palustrine wetlands requires certain ponding environment conditions to maintain soil moisture and humidity, which is difficult to achieve in large slope areas. Floodplain wetlands exist in gentle terrain areas to be able to intercept precipitation and accept floods that overflow the riverbed during the flood season. In the dry season, the recharge water in floodplain wetlands is released to shorten the dry time and maintain the base flow of riverine wetlands, thus regulating the river runoff [45,46].

**Figure 4:**
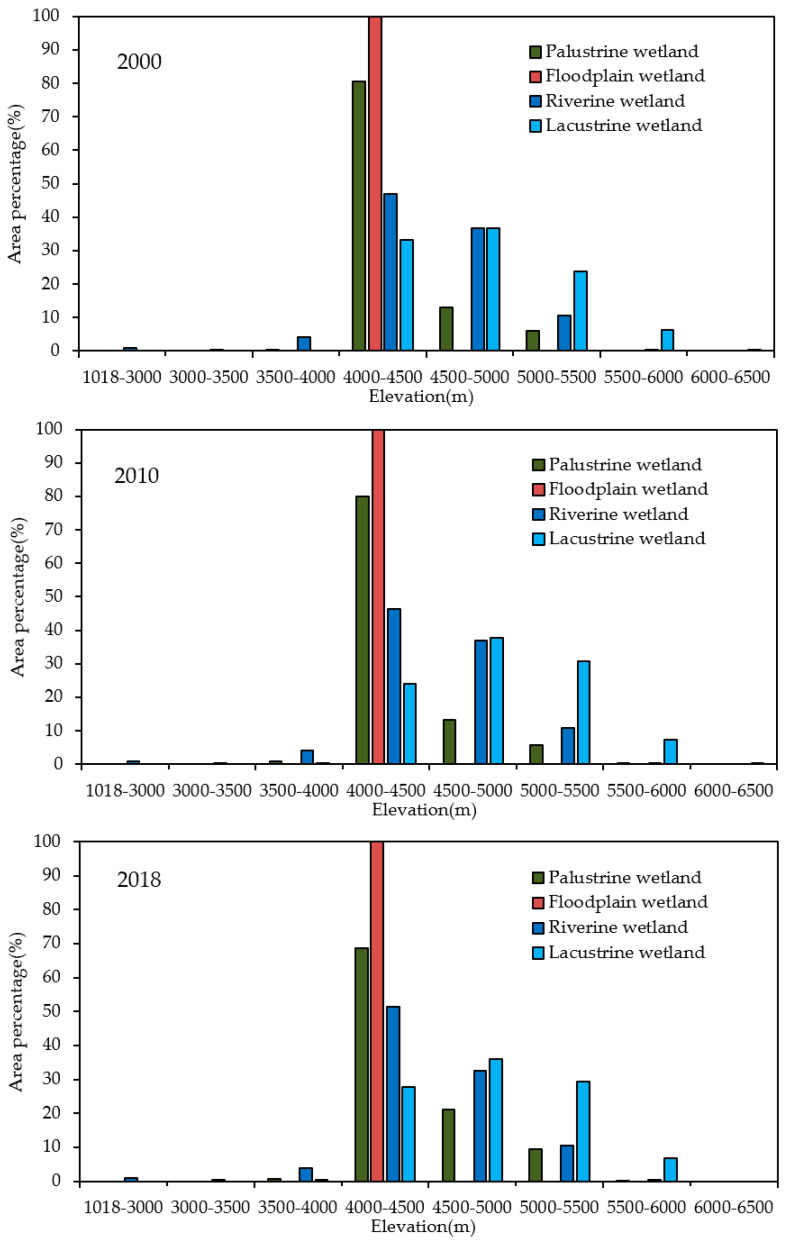
Elevation characteristic of wetlands in the PRB.

The elevation in this basin ranges from 1018 to 8806 m. Influenced by factors such as landform and hydrothermal combination conditions, the wetlands were concentrated in three elevation zones of 4000 to 4500 m, 4500 to 5000 m, and 5000 to 5500 m, accounting for 57.33%, 26.07%, and 13.23% of the total wetland area, respectively. In general, the wetlands were mainly distributed in the areas with elevations between 4000 and 4500 m. There were lacustrine wetlands of 0.10 km^2^ at 6000 to 6500 m in 2000 and 2010, with no wetlands distributed above 6500 m (Figure 4).

In 2000 and 2010, lacustrine wetlands were scarcely distributed at altitudes of 6000 to 6500 m, but the upper limit of lacustrine wetland distribution decreased to 6000 m in 2018. Furthermore, lacustrine wetlands were not distributed at 3500 to 4000 m in 2000, but there was a distribution in 2010 and 2018, and the area proportion in 2018 increased relative to that in 2010. This phenomenon indicates that lacustrine wetlands tend to be distributed at low altitudes. In contrast, the distribution of palustrine wetlands tends to be higher altitudes. In 2010, palustrine wetlands were scarcely distributed at altitudes of 5500 to 6000 m, but in 2000, there was no palustrine wetland distribution. In 2018, the proportion of palustrine wetland distribution in this altitude range increased, and the proportions at altitudes of 4500 to 5000 m and 5000 to 5500 m increased significantly.

#### 3.1.4. Spatial Characteristics of Wetland Changes

**Figure 5:**
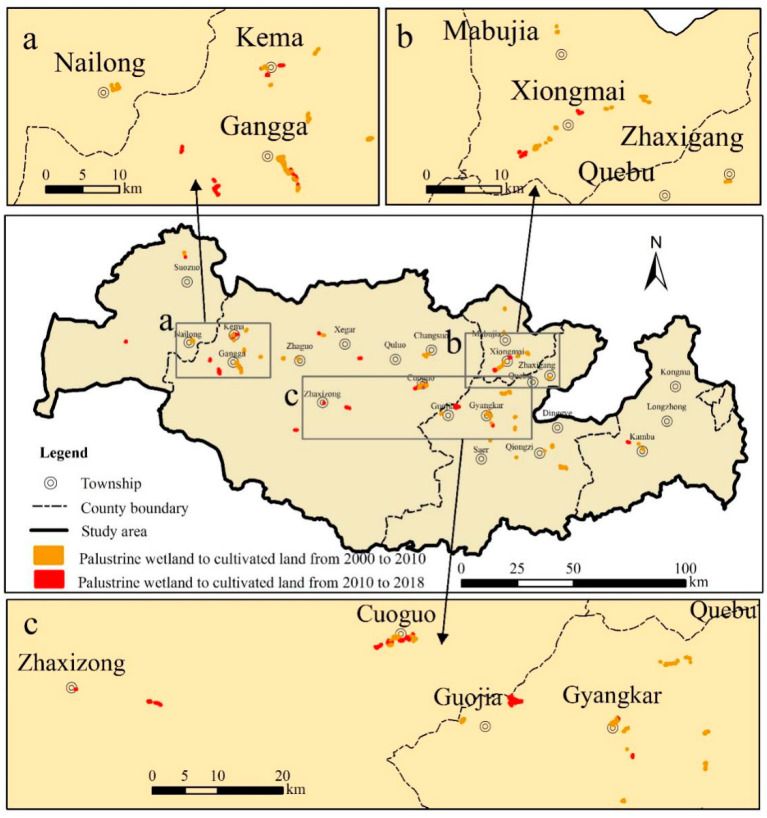
Spatial distribution of cultivated land reclaimed from palustrine wetland in the PRB in different periods. Reclamation near Gangga town of Tingri County (**a**); Reclamation near Xiongmai village of Sa’gya County (**b**); Reclamation near Cuoguo town, Gyangkar town in the middle part (**c**).

The palustrine wetlands were in a state of constant decline. They decreased the most from 2000 to 2010, reaching 17.72 km^2^. The reduction trend from 2010 to 2018 was alleviated, and the reduction area was 5.39 km^2^. From the perspective of space, most of the changes in palustrine wetlands occurred in residential areas or in the vicinity of towns, indicating that human activities influenced palustrine wetland changes to some extent. On the Tibetan Plateau, the most important human activities affecting wetlands are reclamation and overgrazing. Reclamation from palustrine wetlands to cultivated land for grain cultivation was common in the study area, and wetlands were the main sources of new cultivated land. To clarify the specific distribution and area of cultivated land reclaimed from palustrine wetlands, the region of palustrine wetland reduction in 2000–2010 was superimposed with the cultivated land distribution in 2010, and the region of palustrine wetland reduction in 2010–2018 was superimposed with the cultivated land distribution in 2018 to obtain the spatial distribution of cultivated land reclaimed from palustrine wetlands in the PRB (Figure 5). In the past 20 years, reclamation mainly occurred near Gangga town (Old Tingri County town) of Tingri County (Figure 5a), Xiongmai village of Sa’gya County (Figure 5b), Cuoguo town, and Gyangkar town (Dinggye County town) in the middle part (Figure 5c). Our results were consistent with the discovery of Li [47]. In this study, from 2000 to 2010, the cultivated land area in palustrine wetlands was 2.76 km^2^. From 2010 to 2018, the reclamation area of palustrine wetlands was 1.24 km^2^. Compared with the first 10 years, the reclamation area in the last 10 years decreased by more than half. The southern part of the study area was mostly glaciers and glacial lakes, and there were few palustrine wetlands.

**Figure 6:**
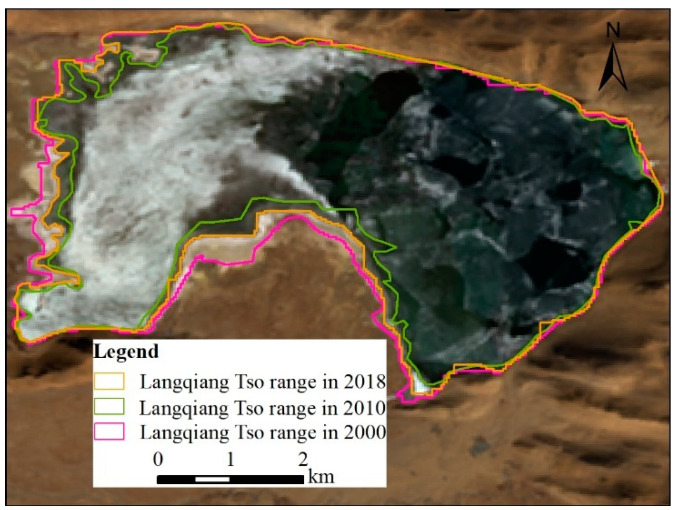
Langqiang Tso water surface change from 2000 to 2018.

During the study period, the riverine wetlands and lacustrine wetlands experienced a process of first decreasing and then increasing. The area decreased from 2000 to 2010 and then increased from 2010 to 2018. The change in riverine wetlands was not apparent, while the lacustrine wetlands had obvious changes. Taking the Langqiang Tso as an example (Figure 6), the area in 2002 was 24.88 km^2^, then decreased to 21.41 km^2^ in 2010, and increased to 23.66 km^2^ in 2018, which was consistent with the overall trend of lacustrine wetlands. 

**Figure 7:**
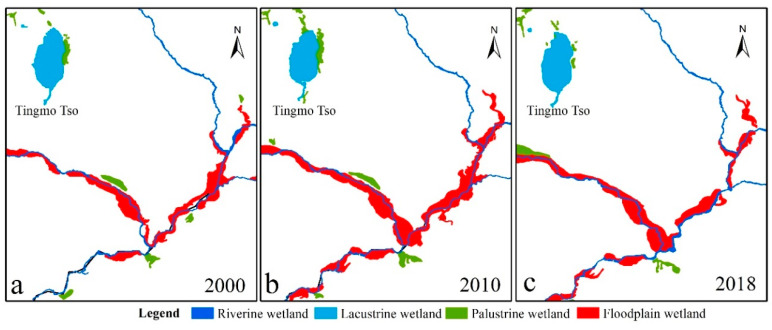
Typical floodplain wetland changes from 2000 to 2018; 2000 (**a**), 2010 (**b**), and 2018 (**c**).

The floodplain wetland changes were opposite to those of the lacustrine wetland, showing a trend of first increasing and then decreasing. From 2000 to 2010, the area increased by 8.24 km^2^, and then decreased by 11.82 km^2^ from 2010 to 2018. The floodplain wetlands were mainly distributed in the central part of the PRB, the two sides of the Tingmo Tso, and extended to the Tingri County boundary. Taking the floodplain wetlands located downstream of Tingmo Tso as an example, in 2000, the floodplain wetlands were distributed along the river and in scattered strips (Figure 7a). In 2010, it was clearly seen that the floodplain wetlands were distributed in blocks on both sides of the river, and the area increased (Figure 7b). In 2018, the area of the upper right floodplain wetlands greatly decreased (Figure 7c).

### 3.2. Relationship between Wetland Changes and Climate Change

**Figure 8:**
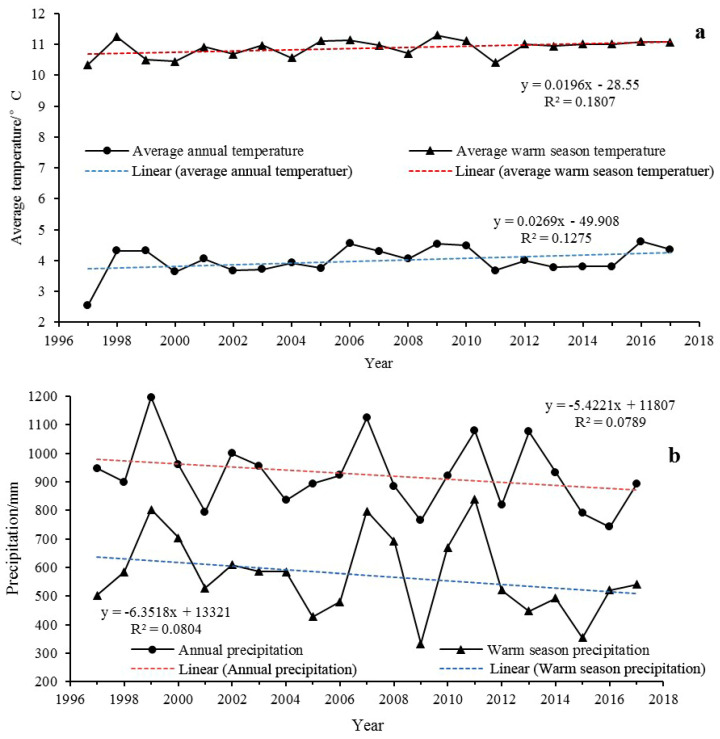
Annual average temperature and warm-season average temperature (**a**), annual precipitation and warm-season precipitation (**b**) change trends of the PRB from 1997 to 2017.

As reported by Liu et al. [48], climate change was one of the main driving factors of wetland changes, and this study focused on the impacts of climate change on wetlands. To show the continuous change trend of temperature and precipitation, the temperature (Figure 8a) and precipitation data (Figure 8b) of the PRB from 1997 to 2017 are as follows.

Grey correlation analysis was conducted to reveal the influence of climate on the wetland area. According to Formulas (1) and (2), we obtained the correlations among riverine wetlands, lacustrine wetlands, palustrine wetlands, floodplain wetlands, and total wetlands and annual average temperature, warm-season average temperature, annual precipitation, and warm-season precipitation (Table 6). The results showed that, except for the correlation between warm-season precipitation and riverine wetlands that was below 0.5, the correlation among other climatic factors and the wetland area was above 0.5, indicating a positive correlation.

#### 3.2.1. Wetland Area and Temperature

The temperature change in the PRB generally increased from the fitting trend line. The change rate of the annual average temperature was 0.27 °C·10 year^−1^. In the past 20 years, the annual average temperature has risen by approximately 0.8 °C. The change rate of the warm-season average temperature was 0.20 °C·10 year^−1^, which was less than that of the annual average temperature. Compared with the annual average temperature, the warm-season average temperature change was relatively stable, showing a steady upward trend. During this period, the total wetland area decreased, which showed a negative correlation between the wetland area and temperature. An increase in temperature will increase wetland water temperature and soil temperature, resulting in an increase in wetland evaporation, thereby affecting wetland changes. Among them, the total wetland area had the largest negative correlation with the warm-season average temperature, with a correlation of 0.9185. The total wetland area had a small correlation with the annual average temperature, which was 0.6586 (Table 6). The warm-season average temperature correlation of each wetland was greater than the annual average temperature. In general, the warm-season average temperature had a more significant impact on the wetland area. The warm-season temperature was the highest in a year, and wetland evaporation also increased sharply, which had the most obvious impact on wetland ecology.

From the perspective of various wetland types, palustrine and floodplain wetlands were negatively correlated with temperature, which was consistent with the total wetland area. Riverine and lacustrine wetlands were positively correlated with temperature. Combined with the distribution of riverine and lacustrine wetlands in the PRB, the impact of rising temperature on riverine and lacustrine wetlands was reflected not only in the negative impact of aggravating the evaporation process but also in glacier retreat and ice and snow melting. These processes supplement the water volume and have a positive impact on riverine and lacustrine wetlands. The influence of evaporation on lacustrine wetlands on the Tibetan Plateau is less than that of glacier retreat and snow melting [49]. For example, the contribution of evaporation to Nam Co was only 4% [50], while the contribution of glacier recharge was approximately 19% [51].

#### 3.2.2. Wetland Area and Precipitation

Contrary to the trend of temperature change, the amount of precipitation showed a downward trend. During the study period, the change rates of annual precipitation and warm-season precipitation were −54.22 mm·10 year^−1^ and −63.5 mm·10 year^−1^, respectively, and the warm-season precipitation decreased more than the annual precipitation. There was a positive correlation between wetland area and precipitation. The increase in precipitation increases the hydrological acquisition of wetlands and directly promotes the expansion of wetland areas. Among them, the wetland area had a large positive correlation with annual precipitation, with a coefficient of 0.7253, and the warm-season precipitation coefficient was relatively small, i.e., 0.5749. Except for floodplain wetlands, the correlation of annual precipitation was greater than that of the warm-season precipitation. In general, the annual precipitation had a more significant impact on the wetland area. 

From the perspective of various wetland types, palustrine and floodplain wetlands were positively correlated with precipitation, which was consistent with the total wetland area. Riverine and lacustrine wetlands were negatively correlated with precipitation. The possible reason was that the hydrological supply of glaciers and snow melting caused by rising temperature offset insufficient precipitation, which increased riverine and lacustrine wetland areas, indicating that the hydrological supply of riverine and lacustrine wetlands in the PRB mainly came from glaciers and snow melting.

#### 3.2.3. Wetlands and Climatic Factors

The correlations between riverine wetlands and temperature and between lacustrine wetlands and temperature were greater than their correlation with precipitation, which further indicated that the hydrological recharge of riverine and lacustrine wetlands mainly came from the melting of glaciers and snow. Riverine wetlands and lacustrine wetlands had the highest correlation with warm-season average temperature, with coefficients of 0.8661 and 0.8524, respectively. The correlation of warm-season average temperature was more than the annual average temperature, indicating that the warm-season temperature had a greater impact on riverine and lacustrine wetlands. The warm-season average temperature is higher than the annual average temperature. In the warm season, the higher temperature increases the melting of glaciers and snow mountains and releases more water than in other months of the year.

Palustrine wetlands had the largest correlation with annual precipitation, which was 0.8324, followed by the warm-season average temperature, with a coefficient of 0.8017. Vegetation exists in palustrine wetlands, and the survival and growth of vegetation are highly dependent on water. Therefore, annual precipitation had the greatest correlation with palustrine wetlands. The warm-season average temperature affected palustrine wetlands in two ways. First, it promoted evaporation of palustrine wetlands and reduced wetland water. Second, the increase in warm-season average temperature introduced more heat, which made conditions more beneficial to mesophytes. When evaporation becomes stronger, mesophyte growth and invasion become the dominant species, the number of hygrophytes decreases and palustrine wetlands gradually degenerate into grasslands [52].

Floodplain wetlands had the highest correlation with warm-season precipitation, with a coefficient of 0.6519. The correlation between floodplain wetlands and precipitation was greater than that of temperature (the correlation of warm-season precipitation was greater than warm-season average temperature, and the correlation of annual precipitation was greater than annual average temperature). Floodplain wetlands are distributed on both sides of riverine wetlands, and there is no fixed water supply; thus, they rely more on precipitation and groundwater.

The total wetland area had the greatest correlation with the warm-season average temperature, followed by the annual precipitation, annual average temperature and warm-season precipitation. The warm-season average temperature had an important influence on riverine, lacustrine and palustrine wetlands through different influence forms.

## 4. Discussion

### 4.1. Wetland Changes

Palustrine wetlands in the PRB decreased by 10.09%. Compared with other parts of the Tibetan Plateau, the reduction was slight. From 1970 to 2010, freshwater marsh and salt marsh on the Tibetan Plateau decreased by 46.6% and 53.9%, respectively [53]. From 2000 to 2015, palustrine wetlands in the Zoige Plateau decreased by 27.55% [41]. From 1969 to 2013, palustrine wetlands in the Yangtze River and Yellow River source area decreased by 45.18% and 54.39%, respectively [54]. Natural factors were the key contributors to wetland changes in the PRB. Although there was a phenomenon of farmers reclaiming palustrine wetlands to cultivated land, the impact intensity on palustrine wetlands in the region was generally small. Wet soil in palustrine wetlands and full hydrological conditions were suitable for plant growth while also meeting the requirements of farming [15]. Good natural conditions make the cost of reclaiming palustrine wetlands far lower than that the of wastelands. From 2000 to 2010, wetlands reclaimed as cultivated land accounted for 15.58% (2.76 km^2^) of the decrease in palustrine wetlands in the PRB. From 2010 to 2018, the proportion was 23.01% (1.24 km^2^). Due to different geographical locations, regional natural and socio-economic conditions are different, the impact on wetland changes varies greatly. Studies have shown that in areas with high population pressure, such as Ethiopia in Africa and Hangzhou Bay in China, reclaiming wetlands is a response to the rapid population growth [55,56]. In the agriculturally developed areas such as Northeast China, reclaiming wetlands is an inevitable choice for agricultural production [17]. Although people in the PRB rely on agriculture, the population density is only approximately one thirty-ninth of China [57], the agricultural GDP is low (China Statistical Yearbook), and the reclamation behavior is relatively small.

Riverine and lacustrine wetland area increased, which was consistent with the change trend of riverine and lacustrine wetlands on the Tibetan Plateau. From 2008 to 2016, riverine wetland area on the Tibetan Plateau increased by 25.5% [58]. From 2000 to 2018, lacustrine wetland area on the Tibetan Plateau expanded by 22.9% [59]. The factors that most affected the riverine and lacustrine wetlands were runoff variation and water level change, respectively [60,61]. Because of the climate change, hydrological recharges and evaporations of riverine and lacustrine wetlands are different, thus affecting the runoff and water level. Affected by different sources, riverine wetland changes are different in regions. Most riverine wetlands on the Tibetan Plateau originate from glaciers, and the amount of water melted by glaciers has a significant impact on river runoff [62]. However, in areas without glaciers, most riverine wetlands originate from high mountains and rely on the terrain to form riverine wetlands. The influencing factors of such riverine wetland changes are relatively single, e.g., Zhujiang River, China [63]. For lacustrine wetlands, there are differences between glacial lakes and non-glacial lakes, and glacial lakes are affected by glaciers also prone to outburst floods [64]. Chongbaxia Tsho glacial lake outburst flood in the Eastern Himalaya originated an ice avalanche from the parent glacier [65].

Floodplain wetland changes greatly, mainly related to riverine and lacustrine wetlands. This result was because the floodplain wetlands in the PRB were around the riverine wetlands, and at the lacustrine wetlands downstream, floodplain wetlands may have a series of hydrological processes associated with the adjacent riverine and lacustrine wetlands on the ground and underground [66,67]. Riverine wetland hydrology changes, and if the frequency and amplitude of runoff scouring are too small, the soil moisture on both sides of the riparian zone will decrease, the groundwater level will decrease, and the vegetation will degrade [68,69]. If the runoff is too large, the riparian soil will be saturated and the living environment will deteriorate, which will destroy the riparian vegetation and reduce the species diversity [70,71]. Floodplain wetlands exist in areas with gentle terrain and intercept floods in the flood seasons. Through hydrological connections, water resources can be released slowly during dry seasons, and the dry-up time of downstream rivers can be shortened to maintain the basic flow of rivers, and achieve the regulation of river runoff [45,46]. From 1988 to 2015, 17.10% of the riverine wetlands in the source region of the Yangtze River have been transformed into floodplain wetlands [22]. In this study, from 2000 to 2010, the Tingmo Tso area decreased from 10.82 km^2^ to 9.37 km^2^, and then increased to 10.94 km^2^ in 2018, which was opposite to the floodplain wetland changes, which may be why part of the water lost by the lacustrine wetlands was kept in the floodplain wetlands.

### 4.2. Climatic Factors Impact Wetlands

Climatic factors play a substantial role in the field of wetland changes. Temperature and precipitation were shown to be the main natural driving forces [72]. The meteorological data of the PRB showed that in the past two decades, the average temperature had increased by approximately 0.8 °C and precipitation had decreased by approximately 100 mm. Compared with the annual average temperature and annual precipitation, the influence of warm-season temperature and precipitation on wetland changes was more critical. In the warm season, some glaciers and snow melt, which can supply the water source of the wetland [73]. The warm-season is also the growing season of wetland vegetation, which shows that temperature and precipitation affect the growth of vegetation. However, climate warming makes vegetation more dependent on water conditions, and annual precipitation plays a key role in vegetation survival. The palustrine wetlands in the study area do not have a stable water supply and rely on replenishment from precipitation. More precipitation leads to the more hydrological recharge of palustrine wetlands. The palustrine wetlands were influenced by precipitation more than temperature changes. Similar to the climate change trend in the PRB, the Zoige Plateau and the Yarlung Zangbo River Basin have increased annual average temperature and decreased annual precipitation [19], but wetland changes had a strong correlation with temperature. Palustrine wetland area in the Zoige Plateau decreased by 56.54% from 1977 to 2016, and the temperature rise directly led to hydrological conditions change of the palustrine wetlands, which was the most important natural driving factor [74]. Palustrine wetlands of the Mcdika Reserve in the Yarlung Zangbo River Basin decreased by 20% from 1988 to 2015, and the temperature and surface temperature increase are the main reasons affecting the degradation of the palustrine wetlands [75].

The river runoff in the study area is mainly composed of glaciers, frozen soil, and groundwater recharge. Most rivers originate from glaciers; for instance, the most important river, the Pumqu River, originates from the Yebokangal Glacier on the northern slope of Mount Shishapangma Peak. In the warm-season, glaciers and frozen soils are greatly affected by temperature. The higher temperature causes the melting of glaciers and frozen soils, which benefit the hydrological conditions of riverine wetlands [76]. The influence of precipitation on the riverine wetlands in the PRB was not apparent, which was consistent with the conclusion of Wang [77]. Among them, the impact of warm-season precipitation was clearly not significant. This result may represent a sharp increase in warm-season temperature, which leads to increased evaporation and offsets the supplement of precipitation. Due to the significant temperature rise, the evapotranspiration consumption of riverine wetlands in the source area of the Yellow River was greater than the replenishment of glaciers and snow melting. Although the precipitation increased during the same period, the riverine wetland runoff in the region decreased from 1955 to 2005 [78]. The source of runoff recharge also indicated that riverine wetlands in the PRB did not depend on precipitation. Liu et al. [79] reported that the temperature in the Yarlung Zangbo River Basin increased by 1.2 °C from 2000 to 2010, riverine wetland area in the region increased the fastest. The temperature rise led to the melting of glaciers and snow, which became an important water source of riverine wetlands. The runoff of the main stream, Niyang River, Yigong River and other tributaries mainly supplied by melt water increased rapidly [79].

There are approximately 75 glacial lakes in southern part of the study area. In the case of climate change, lacustrine wetland changes are more complex and are affected by the expansion of glacial lakes and the shrinkage of nonglacial lakes [80]. The increase in temperature and the decrease in precipitation lead to glacial retreat and glacial lake expansion, but nonglacial lakes, on the other hand, are shrinking. As the largest single lacustrine wetland in the PRB is only 23.66 km^2^ (2018), compared with the large lacustrine wetlands in other regions of the Tibetan Plateau, there are differences in the effects of temperature and precipitation. For the large lacustrine wetlands, the amount of glacier replenishment is limited, and precipitation is the main factor affecting the lacustrine wetland changes. Biskop et al. [51] used hydrological models to study the influencing factors of Mapam Yumco (approximately 412 km^2^) and Paiku Co (approximately 270 km^2^) from 2001 to 2011, and found that the contribution of precipitation accounted for 85% and 70%, respectively, and that of glaciers only accounted for 15% and 30%, respectively. Tang et al. [81] found that Qinghai Lake area (approximately 4400 km^2^) increased by approximately 3% from 2005 to 2016 due to increased precipitation.

Floodplain wetlands are the flat areas on both sides of riverine wetlands, which are flooded and submerged by rivers, and they are most affected by seasonal precipitation. On average, warm-season precipitation accounted for 60% of the annual precipitation in the PRB, and the intercepted precipitation during this period was an important source of water conservation in the floodplain wetlands. 

The analysis of each wetland type shows that the wetland loss in the plateau is reversible. For different wetland types, the conditions and process of wetland restoration were different. Due to the special geographical conditions and climate of the Tibetan Plateau, riverine and lacustrine wetland changes were not only directly affected by the amount of precipitation and evaporation but also influenced by the water supply of melting glaciers and snow. The total area of lakes larger 1 km^2^ on the Tibetan Plateau decreased by 5.6% from 1976 to 1995 but rapidly expanded by 22.9% from 2000 to 2018 [59]. The riverine wetland area in the source region of the Yellow River first decreased and then increased; although the overall area decreased, the change trend gradually improved [21]. From 2000 to 2010, the palustrine wetland area in the Zoige Plateau continued to decrease to 64.75% in 1990, and gradually recovered to 72.45% from 2010 to 2015 [41]. The palustrine wetland changes were related to human activity on the Zoige Plateau [41]. Riverine wetlands in the source region of the Yangtze River have been decreasing, and 17.10% of the riverine wetlands have been transformed into floodplain wetlands [22].

### 4.3. Policy Implications

The main areas of reclamation from wetlands are concentrated around towns. This is because for more than 20 years, the government has vigorously built irrigation and water conservancy facilities in places where the population is concentrated, forming centralized and continuous irrigation ditches. The irrigation conditions of cultivated land have improved and farmers can have more cultivated land. From 1995 to 2015, the cultivated land irrigation area in the PRB increased from 46.30 km^2^ to 89.86 km^2^, with an annual average growth rate of 4.70% [82]. Reclamation has severely damaged the wetland ecological environment. Although reclamation is not the main cause of wetland changes, the reclamation and construction of cultivated land directly damage the ecological environment of the original wetland. However, reclamation indirectly affected the ecological environment of the wetland around the new cultivated land. Different species of cultivated land and wetlands need different nutrients and have different ecological sensitivities. The problem of nonpoint source pollution by agriculture may be transferred to adjacent wetlands, the ecological impact is more intense.

**Figure 9:**
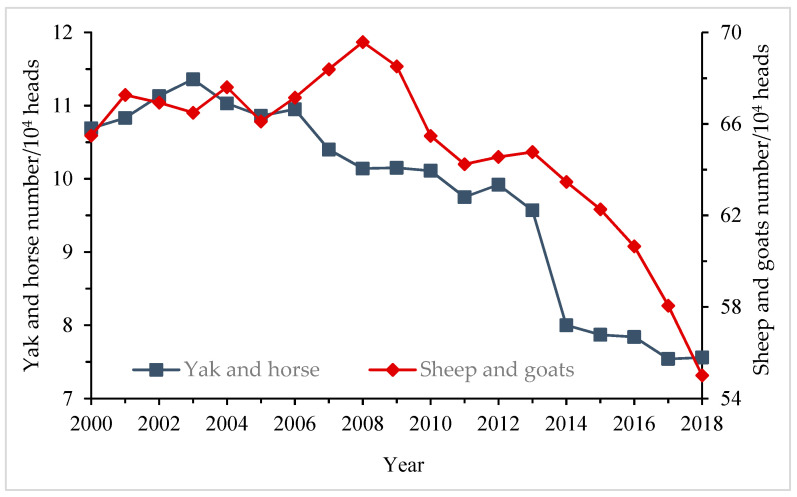
Number changes of yaks and horses, sheep and goats in Tingri, Dinggye, and Nyalam from 2000 to 2018 (Source: Tibet Statistical Yearbook).

In the past 10 years, the situation of wetlands has improved, and the total wetland area has increased slightly. Since the implementation of China’s 12th Five-Year Plan, Tingri, Dinggye and Nyalam Counties have focused on the construction of ecological protection and payment for ecosystem service (PES) projects (http://www.scio.gov.cn/zhzc/8/1/document/1443109/1443109.htm (accessed on 5 February 2021)). In 2015, the Forestry Department of Tibet Autonomous Region of China launched the ecological compensation (pilot) project of important wetlands in Dinggye County of Mt. Everest National Nature Reserve. The government fenced the local wetlands, implemented grazing restrictions and prohibitions, and issued subsidies for the people. In addition to agriculture, animal husbandry is an important part of the national economy in the PRB and is one of the local pillar industries. For a long time, the number of livestock in the PRB was maintained at a relatively high level. Overgrazing accelerates wetland water loss and degrades wetlands. In the past 10 years, the government has been aware of this negative impact and convinced local herders to reduce their livestock (Figure 9). From 2010 to 2018, the total number of yaks and horses, sheep and goats in Tingri, Dinggye, and Nyalam Counties decreased by 25.4% and 11.3%, respectively. The implementation of a series of policies has protected and restored the ecological environment around degraded wetlands, maintaining the stability and sustainability of ecological development. Due to the particularity and importance of alpine wetlands, they are worthy of additional research attention. Therefore, two suggestions can be put forward as follows.

First, the government should actively balance the relationship between wetland protection and the livelihoods of farmers and herdsmen. As a Contracting State of the Ramsar Convention, China has dedicated much money and energy for wetland protection. Meanwhile, to protect the livelihoods of farmers and herdsmen, China has formulated an ecological compensation strategy. Through the implementation and improvement of ecological compensation strategies, the industrial structure of agriculture and animal husbandry can be rationally adjusted. Farmers should change the situation of relying solely on drainage wetlands to increase cultivated land area and grain yield, and herders can reduce grazing activities. At the same time, farmers and herders ought to be guided to engage in nonagricultural activities. Relying on the rich local wetland resources and the “wise use” principle of the Ramsar Convention, within the scope of local resources and environmental carrying capacity, the utilization of wetlands has changed from material use to spiritual use. The government needs to give farmers and herders more skills training, enabling them to engage in secondary and tertiary industries, such as ecotourism. For example, people could develop tourism brands such as the Dinggye Wetland Kingdom, create special handmade souvenirs, establish wetland museums, and carry out educational programs and activities related to wetland environmental protection for tourists during the tourism season. At the same time, corresponding laws and regulations should be formulated to strictly restrict tourists’ unfriendly behaviors towards the environment. Part of the income from ecotourism can be put into wetland protection to sustain of wetland utilization.

Second, some measures should be taken to improve public awareness of wetland protection. Through radio, newspapers, reports, roving exhibitions, and other propaganda media, we can actively carry out publicity and education to raise public awareness of wetland protection. The government of the Tibet Autonomous Region of China has employed poor farmers and herders to manage and protect wetlands in combination with precision-targeted poverty alleviation. Although the government paid money, it could enhance people’s ownership sense of wetland protection, play a better effect on wetland protection, and improve the ecological service value of wetlands. In addition, a series of technical trainings and publicity activities should be carried out for farmers and herders to enhance their awareness and ability to protect wetlands. We should also publicize the deeds of advanced figures in wetland protection, improve people’s inner sense of participation in wetland protection, and let them play an important role in wetland protection. 

### 4.4. Study Limitations

The study results showed that climate change had a significant impact on the wetland changes in the PRB. However, previous studies have shown that in addition to climatic factors, human activities had an impact on wetland changes [83,84,85]. However, unlike the plains and coastal areas, the impact of human activities is very limited due to the relatively low intensity of human activities on the Tibetan Plateau; thus, it has not been studied in depth in this paper. In the future, more research should be conducted to explore the factors affecting wetland changes, and to develop models based on different scenarios to quantify the extent of impact. We could then establish a closer link between the long-term impact of climate change and human activities.

Moreover, future research should evaluate the linkages and feedback mechanisms between hydrology and wetland changes. For instance, the hydrological function between wetlands and other landscape types (such as glaciers) should be found, or the impact of hydrological connectivity among wetland types should be assessed [76,86].

## 5. Conclusions

Wetland changes and its causes are the topic of global environmental change research. Wetlands have the functions of ecological water storage, water supply and climate regulation, which plays an indispensable role in global environmental security. The Tibetan Plateau is undergoing intense climate change, and the rate of climate warming is faster than that in other parts of the world. Wetland is one of the most fragile ecosystems. Climate change and human activities affect wetlands drastically. Once destroyed, it is difficult to restore in a short time. In this paper visual interpretation was applied to Landsat TM/OLI series images from three years (2000, 2010, 2018) to obtain multitemporal wetland datasets to understand the wetland distribution, changes and causes in the PRB. We identified 459.71 km^2^ of wetlands in the PRB in 2018, accounting for 1.84% of the basin area. The wetlands in the basin were mainly palustrine wetlands (44.77%), followed by riverine wetlands (28.88%) and lacustrine wetlands (20.21%). Due to water storage conditions caused by terrain and the hydrothermal conditions caused by elevation are different, wetlands were mainly distributed in areas with slopes less than 12° and at elevations between 4000 and 5500 m. There were strong slope and elevation differentiation among the wetland distribution. The total wetland area showed a downward trend from 2000 to 2018 in the PRB. Palustrine wetlands have been decreasing, but the decreasing trend has improved since 2010. Palustrine wetlands were reclaimed to cultivated land, but the proportion of reclamation is small. Climate dominated wetland changes in the PRB. Affected by climate change, the areas of riverine lacustrine, and floodplain wetlands are in a process of dynamic changes. There are glacial lakes in the PRB, and important riverine wetlands originate from glaciers. The melting of glaciers and snow caused by rising temperature has an important impact on the water supply of riverine and lacustrine wetlands. For sustainable development, the government plays a guiding role and actively formulates and implements wetland protection policies, such as restricting or prohibiting grazing on wetlands, which play an important role in wetland protection and restoration. This research achieved a synthesized evaluation of the wetland distribution, changes, and causes in the PRB, which will benefit sustainable wetland ecosystem management and the understanding of global wetland dynamics in response to climate change.

## Figures and Tables

**Table 1:** Landsat images and their acquisition dates.

Path	Row	Acquisition Date		
139	40	30 November 1999	2 April 2010	7 March 2018
139	41	17 January 2000	13 February 2010	7 March 2018
140	40	24 January 2000	9 April 2010	14 March 2018
140	41	21 November 1999	5 March 2009	9 January 2018
141	40	3 March 2000	9 December 2009	16 January 2018

**Table 2:** Classification system and interpretation keys of alpine wetlands in the PRB.

Classification System of Wetland	Characteristics	Interpretation Keys	Image
Riverine wetland	Permanent or intermittent moving water bodies, excluding floodplain and ait.	It appears as black or blue-black and has a long and narrow curve in images.	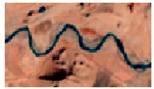
Lacustrine wetland	Under the crustal tectonic movement, glaciation, river erosion, the surface forms concave fields, and the water accumulates into the lake. The lacustrine wetland (including the glacial lake) does not include the surrounding wetlands.	It appears as blue or black in images, with an irregular shape, uniform tone but clear boundaries with the land.	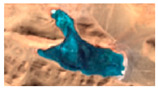
Palustrine wetland	Dominant species are aquatic plants, which are distributed in valleys or low-lying plains, and the wetlands are related to the supply of abundant water such as rivers, glaciers and springs.	It appears as light red and some with light blue or black hydrological marks in images.	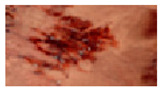
Floodplain wetland	Plain areas on both sides of inundated rivers, including beaches, flooded valleys and flooded grasslands.	It appears as blue-gray, blue-black or brown-red, distributed along the river.	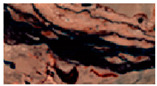

**Table 3:** Wetlands area change in the PRB from 2000 to 2018.

Type	2000		2010		2018		2000–2010		2010–2018		2000–2018	
	km^2^	%	km^2^	%	km^2^	%	km^2^	Change%	km^2^	Change%	km^2^	Change%
Riverine wetland	131.41	0.53	128.83	0.51	132.76	0.53	−2.58	−1.96	3.93	3.05	1.35	1.03
Lacustrine wetland	87.77	0.35	78.21	0.31	92.89	0.37	−9.56	−10.89	14.68	18.77	5.12	5.83
Palustrine wetland	228.94	0.92	211.22	0.84	205.83	0.82	−17.72	−7.74	−5.39	−2.55	−23.11	−10.09
Floodplain wetland	31.81	0.13	40.05	0.16	28.23	0.11	8.24	25.90	−11.82	−29.51	−3.58	−11.25
Total	479.93	1.92	458.31	1.83	459.71	1.84	−21.62	−4.50	1.40	0.31	−20.22	−4.21

Note: Change % is the percentage of the change area divided by the initial area of this period.

**Table 4:** Wetland rate in different counties.

	Nyalam	Tingri	Dinggye	Gamba	Sa’gya
Riverine wetland	0.81%	0.57%	0.36%	0.38%	0.42%
Lacustrine wetland	0.84%	0.30%	0.45%	0.10%	0.00%
Palustrine wetland	1.01%	0.58%	0.99%	1.19%	0.74%
Floodplain wetland	0.00%	0.19%	0.12%	0.00%	0.00%

Note: Wetland rate is in the unit area, wetlands account for proportion in land area.

**Table 5:** Slope grade division.

Grades	1	2	3	4	5	6	7	8	9	10	11
Slope (°)	0–3	3–5	5–8	8–12	12–15	15–18	18–21	21–25	25–30	30–35	>35

**Table 6:** The grey correlation between wetland area and climatic factors in the PRB.

Climatic Factors	Riverine Wetland	Lacustrine Wetland	Palustrine Wetland	Floodplain Wetland	Total
Annual average temperature	0.6153	0.7610	0.5937	0.5546	0.6586
Warm-season average temperature	0.8661	0.8524	0.8017	0.5844	0.9185
Annual precipitation	0.5528	0.6494	0.8324	0.5710	0.7253
Warm-season precipitation	0.4454	0.5821	0.6675	0.6519	0.5749

## Data Availability

The data presented in this study are available on request from the first author.

## References

[1] Brinson M.M., Malvarez A.I. (2002). Temperate freshwater wetlands: Types, status, and threats. Environ. Conserv..

[2] Guo M., Li J., Sheng C., Xu J., Wu L. (2017). A Review of Wetland Remote Sensing. Sensors.

[3] Mahdavi S., Salehi B., Granger J., Amani M., Brisco B., Huang W. (2018). Remote sensing for wetland classification: A comprehensive review. GISci. Remote Sens..

[4] Asselen S.V., Verburg P.H., Vermaat J.E., Janse J.H. (2013). Drivers of Wetland Conversion: A Global Meta-Analysis. PLoS ONE.

[5] Davidson N.C. (2014). How much wetland has the world lost? Long-term and recent trends in global wetland area. Mar. Freshw. Res..

[6] Junk W.J., An S., Finlayson C.M., Gopal B., Květ J., Mitchell S.A., Mitsch W.J., Robarts R.D. (2013). Current state of knowledge regarding the world’s wetlands and their future under global climate change: A synthesis. Aquat. Sci..

[7] Reis V., Hermoso V., Hamilton S.K., Ward D., Fluet-Chouinard E., Lehner B., Linke S. (2017). A Global Assessment of Inland Wetland Conservation Status. Bioscience.

[8] Hu S., Niu Z., Chen Y., Li L., Zhang H. (2017). Global wetlands: Potential distribution, wetland loss, and status. Sci. Total Environ..

[9] Ribeiro De Almeida T.I., Penatti N.C., Ferreira L.G., Arantes A.E., Do Amaral C.H. (2015). Principal component analysis applied to a time series of MODIS images: The spatio-temporal variability of the Pantanal wetland, Brazil. Wetl. Ecol. Manag..

[10] Libonati R., DaCamara C.C., Peres L.F., Sander De Carvalho L.A., Garcia L.C. (2020). Rescue Brazil’s burning Pantanal wetlands. Nature.

[11] Guerra A., Roque F.D.O., Garcia L.C., Ochoa-Quintero J.M., Oliveira P.T.S.D., Guariento R.D., Rosa I.M.D. (2020). Drivers and projections of vegetation loss in the Pantanal and surrounding ecosystems. Land Use Policy.

[12] Li X., Song K., Liu G. (2020). Wetland Fire Scar Monitoring and Its Response to Changes of the Pantanal Wetland. Sensors.

[13] Mitsch W.J., Hernandez M.E. (2013). Landscape and climate change threats to wetlands of North and Central America. Aquat. Sci..

[14] Mao D., Wang Z., Du B., Li L., Tian Y., Jia M., Zeng Y., Song K., Jiang M., Wang Y. (2020). National wetland mapping in China: A new product resulting from object-based and hierarchical classification of Landsat 8 OLI images. ISPRS J. Photogramm. Remote Sens..

[15] Mao D., Luo L., Wang Z., Wilson M.C., Zeng Y., Wu B., Wu J. (2018). Conversions between natural wetlands and farmland in China: A multiscale geospatial analysis. Sci. Total Environ..

[16] Yu X., Ding S., Zou Y., Xue Z., Lyu X., Wang G. (2018). Review of Rapid Transformation of Floodplain Wetlands in Northeast China: Roles of Human Development and Global Environmental Change. Chin. Geogr. Sci..

[17] Mao D., Tian Y., Wang Z., Jia M., Du J., Song C. (2021). Wetland changes in the Amur River Basin: Differing trends and proximate causes on the Chinese and Russian sides. J. Environ. Manag..

[18] Ma C., He Y. (2021). Spatiotemporal Trends and Ecological Determinants in Population by Elevation in China Since 1990. Chin. Geogr. Sci..

[19] Kuang X., Jiao J.J. (2016). Review on climate change on the Tibetan Plateau during the last half century. J. Geophys. Res. Atmos..

[20] Wang R., He M., Niu Z. (2020). Responses of Alpine Wetlands to Climate Changes on the Qinghai-Tibetan Plateau Based on Remote Sensing. Chin. Geogr. Sci..

[21] Li X., Xue Z., Gao J. (2016). Dynamic Changes of Plateau Wetlands in Madou County, the Yellow River Source Zone of China: 1990–2013. Wetlands.

[22] Zhao Z., Liu L., Wang Z., Zhang Y., Li L., Liu F. (2020). Dynamic Changes of Plateau Wetlands in the Damqu River Basin, Yangtze River Source Region, China, 1988–2015. Wetlands.

[23] Jiang W., Lv J., Wang C., Chen Z., Liu Y. (2017). Marsh wetland degradation risk assessment and change analysis: A case study in the Zoige Plateau, China. Ecol. Indic..

[24] Qiu P., Wu N., Luo P., Wang Z., Li M. (2009). Analysis of dynamics and driving factors of wetland landscape in Zoige, Eastern Qinghai-Tibetan Plateau. J Mt. Sci..

[25] Dong Z., Hu G., Yan C., Wang W., Lu J. (2010). Aeolian desertification and its causes in the Zoige Plateau of China’s Qinghai–Tibetan Plateau. Environ. Earth Sci..

[26] Li Z., Wang Z., Brierley G., Nicoll T., Pan B., Li Y. (2015). Shrinkage of the Ruoergai Swamp and changes to landscape connectivity, Qinghai-Tibet Plateau. Catena.

[27] Finlayson C.M., Capon S.J., Rissik D., Pittock J., Fisk G., Davidson N.C., Bodmin K.A., Papas P., Robertson H.A., Schallenberg M. (2017). Policy considerations for managing wetlands under a changing climate. Mar. Freshw. Res..

[28] Peimer A.W., Krzywicka A.E., Cohen D.B., Van den Bosch K., Buxton V.L., Stevenson N.A., Matthews J.W. (2017). National-Level Wetland Policy Specificity and Goals Vary According to Political and Economic Indicators. Environ. Manag..

[29] Fickas K.C., Cohen W.B., Yang Z. (2016). Landsat-based monitoring of annual wetland change in the Willamette Valley of Oregon, USA from 1972 to 2012. Wetl. Ecol. Manag..

[30] Zhang W., Zhang Y., Wang Z., Ding M., Yang X., Lin X., Liu L. (2007). Vegetation change in the Mt. Qomolangma Nature Reserve from 1981 to 2001. J. Geogr. Sci..

[31] Salerno F., Guyennon N., Thakuri S., Viviano G., Romano E., Vuillermoz E., Cristofanelli P., Stocchi P., Agrillo G., Ma Y. (2015). Weak precipitation, warm winters and springs impact glaciers of south slopes of Mt. Everest (central Himalaya) in the last 2 decades (1994–2013). Cryosphere.

[32] Han X., Guo Y., Wen L., Mi C. (2015). New Black-necked Crane Grus nigricollis subpopulation recorded in southern Tibet, China. Forktail.

[33] Wei D., Zhao H., Huang L., Qi Y., Wang X. (2020). Feedbacks of Alpine Wetlands on the Tibetan Plateau to the Atmosphere. Wetlands.

[34] Muro J., Canty M., Conradsen K., Hüttich C., Nielsen A., Skriver H., Remy F., Strauch A., Thonfeld F., Menz G. (2016). Short-Term Change Detection in Wetlands Using Sentinel-1 Time Series. Remote Sens..

[35] Tong L., Xu X., Fu Y., Li S. (2014). Wetland Changes and Their Responses to Climate Change in the “Three-River Headwaters” Region of China since the 1990s. Energies.

[36] Zhang J., Zhang Y., Liu L., Ding M., Zhang X. (2011). Identifying Alpine Wetlands in the Damqu River Basin in the Source Area of the Yangtze River Using Object-based Classification Method. J. Resour. Ecol..

[37] Zhang Y., Wang C., Bai W., Wang Z., Tu Y., Yangjaen D.G. (2010). Alpine wetlands in the Lhasa River Basin, China. J. Geogr. Sci..

[38] Nie Y., Li A. (2011). Assessment of Alpine Wetland Dynamics from 1976–2006 in the Vicinity of Mount Everest. Wetlands.

[39] Fang L., Dong B., Wang C., Yang F., Cui Y., Xu W., Peng L., Wang Y., Li H. (2020). Research on the influence of land use change to habitat of cranes in Shengjin Lake wetland. Environ. Sci. Pollut. Res..

[40] Li W., Xue P., Liu C., Yan H., Zhu G., Cao Y. (2020). Monitoring and Landscape Dynamic Analysis of Alpine Wetland Area Based on Multiple Algorithms: A Case Study of Zoige Plateau. Sensors.

[41] Shen G., Yang X., Jin Y., Xu B., Zhou Q. (2019). Remote sensing and evaluation of the wetland ecological degradation process of the Zoige Plateau Wetland in China. Ecol. Indic..

[42] Wang X., Zhang F., Kung H., Johnson V.C. (2018). New methods for improving the remote sensing estimation of soil or-ganic matter content (SOMC) in the Ebinur Lake Wetland National Nature Reserve (ELWNNR) in northwest China. Remote Sens. Environ..

[43] Li F., Chang G., Xiao J., Zhou B., Fu Y. (2009). Relationship between Wetlands Changes and Climate Change in the Yellow River Source Region. J. Nat. Resour..

[44] Kundu P. (2020). Geomorphic control on wetland classification: A case study in Himalayan Floodplain region. Spat. Inf. Res..

[45] Chembolu V., Dubey A.K., Gupta P.K., Dutta S., Singh R.P. (2019). Application of satellite altimetry in understanding river-wetland flow interactions of Kosi river. J. Earth Syst. Sci..

[46] Larocque M., Biron P.M., Buffin-Bélanger T., Needelman M., Cloutier C., McKenzie J.M. (2016). Role of the geomorphic setting in controlling groundwater-surface water exchanges in riverine wetlands: A case study from two southern Québec rivers (Canada). Can. Water Resour. J..

[47] Li X. (2013). Analysis on Landscape Pattern Changes of Typical Alpine Wetland in Kong Ga Town of Ding Ri in Recent 20 Years. Master’s Thesis.

[48] Liu Y., Sheng L., Liu J. (2015). Impact of wetland change on local climate in semi-arid zone of Northeast China. Chin. Geogr. Sci..

[49] Zhang G., Yao T., Xie H., Yang K., Zhu L., Shum C.K., Bolch T., Yi S., Allen S., Jiang L. (2020). Response of Tibetan Plateau lakes to climate change: Trends, patterns, and mechanisms. Earth Sci. Rev..

[50] Ma N., Szilagyi J., Niu G., Zhang Y., Zhang T., Wang B., Wu Y. (2016). Evaporation variability of Nam Co Lake in the Tibetan Plateau and its role in recent rapid lake expansion. J. Hydrol..

[51] Biskop S., Maussion F., Krause P., Fink M. (2016). Differences in the water-balance components of four lakes in the south-ern-central Tibetan Plateau. Hydrol. Earth Syst. Sci..

[52] Xiang S., Guo R., Wu N., Sun S. (2009). Current status and future prospects of Zoige Marsh in Eastern Qinghai-Tibet Plateau. Ecol. Eng..

[53] Xue Z., Lyu X., Chen Z., Zhang Z., Jiang M., Zhang K., Lyu Y. (2018). Spatial and Temporal Changes of Wetlands on the Qinghai-Tibetan Plateau from the 1970s to 2010s. Chin. Geogr. Sci..

[54] Du J., Wang G., Yang Y., Zhang T., Mao T. (2015). Temporal and spatial variation of the distributive patterns and driving force analysis in the Yangtze River and Yellow River source regions wetland. Acta Ecol. Sin..

[55] Hussien K., Demissie B., Meaza H. (2018). Spatiotemporal wetland changes and their threats in North Central Ethiopian Highlands. Singap. J. Trop. Geogr..

[56] Li N., Li L., Lu D., Zhang Y., Wu M. (2019). Detection of coastal wetland change in China: A case study in Hangzhou Bay. Wetl. Ecol. Manag..

[57] Xu X. (2017). Data set of population spatial distribution kilometer grid in China. Data registration and publishing system of data center of resources and environment science. Chin. Acad. Sci..

[58] Lang Q., Niu Z., Hong X., Yang X. (2021). Remote Sensing Monitoring and Change Analysis of Wetlands in the Tibetan Plateau. Geomat. Inf. Sci. Wuhan Univ..

[59] Zhang G., Luo W., Chen W., Zheng G. (2019). A robust but variable lake expansion on the Tibetan Plateau. Sci. Bull..

[60] Zhang G., Xie H., Kang S., Yi D., Ackley S.F. (2011). Monitoring lake level changes on the Tibetan Plateau using ICESat altimetry data (2003–2009). Remote Sens. Environ..

[61] Sorg A., Bolch T., Stoffel M., Solomina O., Beniston M. (2012). Climate change impacts on glaciers and runoff in Tien Shan (Central Asia). Nat. Clim. Chang..

[62] Yao T., Thompson L., Yang W., Yu W., Gao Y., Guo X., Yang X., Duan K., Zhao H., Xu B. (2012). Different glacier status with atmospheric circulations in Tibetan Plateau and surroundings. Nat. Clim. Chang..

[63] Wen L., Wang J., Zhang S., Li J., Liu L. (2018). Remote Sensing Monitoring and Dynamics of Wetland in Zhujiangyuan Nature Reserve. J. Southwest For. Univ..

[64] Nie Y., Sheng Y., Liu Q., Liu L., Liu S., Zhang Y., Song C. (2017). A regional-scale assessment of Himalayan glacial lake changes using satellite observations from 1990 to 2015. Remote Sens. Environ..

[65] Nie Y., Liu W., Liu Q., Hu X., Westoby M.J. (2020). Reconstructing the Chongbaxia Tsho glacial lake outburst flood in the Eastern Himalaya: Evolution, process and impacts. Geomorphology.

[66] Karim F., Kinsey-Henderson A., Wallace J., Godfrey P., Arthington A.H., Pearson R.G. (2014). Modelling hydrological connectivity of tropical floodplain wetlands via a combined natural and artificial stream network. Hydrol. Process..

[67] Robinson S.J., Souter N.J., Bean N.G., Ross J.V., Thompson R.M., Bjornsson K.T. (2015). Statistical description of wetland hydrological connectivity to the River Murray in South Australia under both natural and regulated conditions. J. Hydrol..

[68] Nilsson C., Jansson R., Zinko U. (1997). Long-Term Responses of River-Margin Vegetation to Water-Level Regulation. Science.

[69] Stromberg J.C., Beauchamp V.B., Dixon M.D., Lite S.J., Paradzick C. (2007). Importance of low-flow and high-flow characteristics to restoration of riparian vegetation along rivers in arid south-western United States. Freshw. Biol..

[70] Hawkins C., Bartz K., Neale C. (1997). Vulnerability of riparian vegetation to catastrophic flooding: Implications for riparian restoration. Restor. Ecol..

[71] Rood S.B., Gourley C.R., Ammon E.M., Heki L.G., Klotz J.R., Morrison M.L., Mosley D., Scoppettone G.G., Swanson S., Wagner P.L. (2003). Flows for Floodplain Forests: A Successful Riparian Resto-ration. Bioscience.

[72] Jiang P., Cheng L., Li M., Zhao R., Huang Q. (2014). Analysis of landscape fragmentation processes and driving forces in wetlands in arid areas: A case study of the middle reaches of the Heihe River, China. Ecol. Indic..

[73] Zhang M., Zhao Y., Liu F., Pan X. (2012). Glacier Dynamics and Water Balance in the Qinghai-Tibet Plateau. Environ. Sci. Technol..

[74] Dong L., Yang W., Zhang K., Zhen S., Cheng X., Wu L. (2020). Study of marsh wetland landscape pattern evolution on the Zoigê Plateau due to natural/human dual-effects. PeerJ.

[75] Li Y., Wang J., Shui Y., Chen X., Zheng G., Liu W., Bao X., Wang T. (2018). Analysis of landscape pattern and ecological service function of the Mcdika wetland reserve. Acta Ecol. Sin..

[76] Polk M.H., Young K.R., Baraer M., Mark B.G., McKenzie J.M., Bury J., Carey M. (2017). Exploring hydrologic connections between tropical mountain wetlands and glacier recession in Peru’s Cordillera Blanca. Appl. Geogr..

[77] Wang J. (1990). Preliminary Study of the Meteorologic and Hydrological Characteristics in the Pumqu River, the North Slope of Himalayas. J. Glaciol. Geocryol..

[78] Chang G., Li L., Zhu X., Wang Z., Xiao J., Li F. (2007). Changes and Influencing Factors of Surface Water Resources in the Source Region of the Yellow River. Acta Geogr. Sin..

[79] Liu D., Wang T., Shen W., Lin N., Zou C. (2016). Dynamic of the alpine wetlands and its response to climate change in the Yarlung Zangbo River Valley in recent 30 years. J. Ecol. Rural Environ..

[80] Zhang G., Yao T., Piao S., Bolch T., Xie H., Chen D., Gao Y., O’Reilly C.M., Shum C.K., Yang K. (2017). Extensive and drastically different alpine lake changes on Asia’s high plateaus during the past four decades. Geophys. Res. Lett..

[81] Tang L., Duan X., Kong F., Zhang F., Zheng Y., Li Z., Mei Y., Zhao Y., Hu S. (2018). Influences of climate change on area variation of Qinghai Lake on Qinghai-Tibetan Plateau since 1980s. Sci. Rep..

[82] Wang T., Yan J., Cheng X., Yu Y. (2020). Irrigation Influencing Farmers’ Perceptions of Temperature and Precipitation: A Comparative Study of Two Regions of the Tibetan Plateau. Sustainability.

[83] Song K., Wang Z., Li L., Tedesco L., Li F., Jin C., Du J. (2012). Wetlands shrinkage, fragmentation and their links to agriculture in the Muleng-Xingkai Plain, China. J. Environ. Manag..

[84] Cui B., He Q., Gu B., Bai J., Liu X. (2016). China’s Coastal Wetlands: Understanding Environmental Changes and Human Impacts for Management and Conservation. Wetlands.

[85] Chen H., Zhang W., Gao H., Nie N. (2018). Climate Change and Anthropogenic Impacts on Wetland and Agriculture in the Songnen and Sanjiang Plain, Northeast China. Remote Sens..

[86] Treichler D., Kääb A., Salzmann N., Xu C. (2019). Recent glacier and lake changes in High Mountain Asia and their relation to precipitation changes. Cryosphere.

